# Endothelial TAZ inhibits capillarization of liver sinusoidal endothelium and damage-induced liver fibrosis *via* nitric oxide production

**DOI:** 10.7150/thno.83714

**Published:** 2023-07-16

**Authors:** Jun-Ha Hwang, Woong Heo, Jung Il Park, Kyung Min Kim, Ho Taek Oh, Gi Don Yoo, Jeekeon Park, Somin Shin, Youjin Do, Mi Gyeong Jeong, Eun Sook Hwang, Jeong-Ho Hong

**Affiliations:** 1 Division of Life Sciences, Korea University, Seoul 02841, Korea.; 2 College of Pharmacy and Graduate School of Pharmaceutical Sciences, Ewha Womans University, Seoul 03760, Korea.

**Keywords:** Endothelial dysfunction, Liver fibrosis, Nitric oxide, Liver sinusoidal endothelial cells, Endothelial nitric oxide synthase

## Abstract

**Background:** Endothelial dysfunction is a systemic disorder and is involved in the pathogenesis of several human diseases. Hemodynamic shear stress plays an important role in vascular homeostasis including nitric oxide (NO) production. Impairment of NO production in endothelial cells stimulates the capillarization of liver sinusoidal endothelial cells, followed by hepatic stellate cell activation, inducing liver fibrosis. However, the detailed mechanism underlying NO production is not well understood. In hepatocytes, transcriptional co-activator with PDZ-binding motif (TAZ) has been reported to be involved in liver fibrosis. However, the role of endothelial TAZ in liver fibrosis has not been investigated. In this study, we uncovered the role TAZ in endothelial cell NO production, and its subsequent effects on liver fibrosis.

**Methods:** TAZ-floxed mice were crossed with Tie2-cre transgenic mice, to generate endothelium-specific TAZ-knockout (eKO) mice. To induce liver damage, a 3,5-diethoxycarboncyl-1,4-dihydrocollidine, methionine-choline-deficient diet, or partial hepatectomy was applied. Liver fibrosis and endothelial dysfunction were analyzed in wild-type and eKO mice after liver damage. In addition, liver sinusoidal endothelial cell (LSEC) was used for *in vitro* assays of protein and mRNA levels. To study transcriptional regulation, chromatin immunoprecipitation and luciferase reporter assays were performed.

**Results:** In liver of eKO mice, LSEC capillarization was observed, evidenced by loss of fenestrae and decreased LSEC-specific marker gene expression. LSEC capillarization of eKO mouse is caused by downregulation of endothelial nitric oxide synthase expression and subsequent decrease in NO concentration, which is transcriptionally regulated by TAZ-KLF2 binding to Nos3 promoter. Diminished NO concentration by TAZ knockout in endothelium accelerates liver fibrosis induced by liver damages.

**Conclusions:** Endothelial TAZ inhibits damage-induced liver fibrosis *via* NO production. This highlights an unappreciated role of TAZ in vascular health and liver diseases.

## Introduction

Liver sinusoidal endothelial cells (LSECs) are a specialized vascular cell type located between the sinusoidal lumen and the space of Disse, which play a role in the clearance of blood-derived waste 1, 2. The structure of LSECs differs from that of other endothelial cells. LSECs contain fenestrae, which are open pores with diameters ranging from 100 to 150 nm 3. The fenestrae of LSEC function as fluid-filtering sieves between the space of Disse and the sinusoidal lumen 2. Upon various damaging cues, such as oxidative stress and decreased nitric oxide (NO) bioavailability, LSECs undergo endothelial dysfunction, characterized by defenestration and formation of an organized basement membrane, which is called capillarization 2, 4. The capillarized sinusoid induces hepatic stellate cell activation following liver fibrosis 2.

NO is a gaseous cellular signaling factor that mediates vasodilation. It is synthesized from L-arginine by nitric oxide synthase (NOS), and relays signals to smooth muscles, which surround blood vessels, relax them and induce vasodilation, and attenuate the elevation of blood pressure induced by means of increased blood flow 5. Vasodilation is important for the regulation of blood pressure, and dysregulation of vasodilation is involved in chronic kidney disease and cardiovascular disease 6. NO is induced by laminar blood flow, through the activation of protein kinase B (PKB, also known as Akt) and calcium signaling, which activate NOS enzymes and upregulate NO synthesis 7, 8. Shear stress also stimulates Nos3 gene transcription 9. Recently, reduced NO bioavailability has been shown to evoke endothelial dysfunction in LSECs, through induction of oxidative injury after acute liver damage 10. However, the link between NO bioavailability-induced liver fibrosis and other upstream factors remains poorly understood.

Transcriptional co-activator with PDZ-binding motif (TAZ) and Yes-associated protein (YAP) are transcriptional co-regulators that regulate the transcription of target genes, by binding to various transcription factors. TAZ/YAP activity is regulated by upstream signaling cascades, such as Hippo, Wnt, G-protein-coupled receptor, and mechanotransduction 11-13. The Hippo signaling pathway consists of several components that regulate cell growth and control organ size. In response to various stimuli, mammalian STE20-like protein kinase 1/2 (MST1/2), which forms a complex with the adaptor protein salvador homologue 1 (SAV1, WW45), phosphorylates large tumor suppressor kinases 1/2 (LATS1/2). LATS1/2-interacting protein MOB kinase activator 1 (MOB1). The phosphorylated LATS1/2, together with MOB1, phosphorylate TAZ/YAP. The phosphorylated YAP/TAZ induces the binding of 14-3-3 proteins to sequester them in the cytosol and ultimately subject them to ubiquitin-mediated proteolytic degradation. However, in the inactive state of Hippo signal, the YAP/TAZ translocate to the nucleus and interact with several transcription factors including TEA domain transcription factors (TEAD). The YAP/TAZ-TEAD complexes stimulate their target genes including pro-fibrogenic genes such as connective tissue growth factor (CTGF) and cysteine-rich angiogenic inducer 61 (Cyr61) 11-13.

Hepatocyte-specific YAP/TAZ knockouts has been reported to decrease fibrosis after CCl_4_ injury 14. TAZ has been shown to promote the transition from steatosis to NASH by inducing inflammation and fibrosis *via* induction of Indian hedgehog signaling 15. Silencing of hepatocyte TAZ has been reported to prevent and reverse fibrosis in a murine model of non-alcoholic steatohepatitis (NASH) 16. In contrast, TAZ-knockout in hepatocytes has been shown to induce fibrosis after two-thirds partial hepatectomy (PHx) 17. Additionally, TAZ/YAP regulates endothelial cell proliferation and metabolism during sprouting angiogenesis 18. Laminar shear stress induces TAZ activation and upregulates mesenchymal stem cell osteogenic differentiation 19. However, the role of TAZ in LSECs is not yet well understood.

In this study, we demonstrated that loss of endothelial TAZ promotes endothelial dysfunction in LSECs, which induces downregulation of endothelial nitric oxide synthase (eNOS) expression and NO production in LSECs. Furthermore, we observed that the interaction between TAZ and Krüppel-like factor 2 (KLF2) induces eNOS expression. Finally, we investigated whether the TAZ in LSECs protects them against damage-induced liver fibrosis.

## Materials and Methods

### Mice

Mice carrying the floxed Taz allele 20 were crossed with Tie2-cre mice 21 to generate endothelium-specific TAZ-knockout (eKO) mice. Cre recombinase was expressed under the control of the Tie2-Cre allele and the exon 2 region of the Taz allele was excised. Tie2-Cre mice [B6.Cg-Tg(Tek-cre)1Ywa/J] were purchased from the Jackson Laboratory (Bar Harbor, ME, USA). To induce liver fibrosis, mice were fed a 3,5-diethoxycarbonyl-1,4-dihydrocollidine (DDC) diet for two weeks and a normal chow diet for two weeks. For the methionine/choline deficient (MCD) diet, mice were fed the MCD diet for four weeks. The experimental procedures and animal care protocols were approved by the Institutional Animal Care and Use Committee of Korea University (KUIACUC-2021-0016).

### Drug administration

To activate soluble guanylate cyclase, mice were administered with 1 mg/kg of cinaciguat (BAY 58-2667) by means of oral gavage, or its vehicle (transcutol/cremophor/distilled water, 1:2:7, v/v). Cinaciguat (SML1532), transcutol (537616), and cremophor (C5135) were purchased from Sigma-Aldrich (St. Louis, MO, USA).

### Cell culture

MILE SVEN 1 (MS-1) cells and HEK293T cells were cultured in Dulbecco's modified Eagle's medium with 1% of penicillin/streptomycin supplemented with 5% or 10% fetal bovine serum, respectively. Under all conditions, the cells were incubated at 37 °C, in a 5% CO_2_ containing atmosphere.

### Isolation of liver sinusoidal endothelial cells

Mouse liver sinusoidal endothelial cells (LSECs) were isolated from eight to ten-week-old mice by collagenase perfusion method as described previously 22. Briefly, collagenase IV-perfused mouse liver was shaken in buffer to dissociate cells. Cell-containing buffer was filtered by 40 μm Corning® cell strainer (CLS431750, Merck, Kenilworth, NJ, USA). Then, non-parenchymal cell (NPC) population was isolated by centrifugation at 25 × g. Supernatant NPC fraction was separated, pelleted by centrifugation at 300 × g. NPC pellet was re-suspended in culture media and placed on top of percoll gradient cusion (50% and 25% percoll). After centrifugation at 900 × g for 25 min, LSECs were collected at the interface between 50% and 25% percoll.

### Antibodies

Antibodies against Lyve-1 (ab14917), von Willebrand factor (ab6994), eNOS (ab76198), anti-KLF2 (ab194486), Tead4 (ab97460), and alpha-smooth muscle actin (ab5694) were purchased from Abcam (Cambridge, UK). Anti-Cd34 (14-0341-82) antibody was purchased from Invitrogen (Carlsbad, CA, USA). Anti-TAZ/YAP (8418), anti-YAP (4912), anti-TAZ (83669), anti-iNOS (2982) and anti-Vinculin (4650) antibodies were purchased from Cell Signaling Technology (Danvers, MA, USA). Anti-FLAG (F1804) antibody was purchased from Sigma-Aldrich. Anti-CTGF (sc25440) antibody was purchased from Santa Cruz biotechnology (Dallas, TX, USA). For immunostaining of paraffin-embedded tissues, the primary antibodies were diluted in a ratio of 1:100. For the immunoblot assay, the antibodies were diluted in a ratio of 1:1000. For chromatin immunoprecipitation (ChIP), the antibodies were diluted to a final concentration of 0.5 μg of antibody per μg of DNA.

### Gene knockdown by retroviral transduction

Retroviral vectors and virus particle-packaging vectors were transfected into Phoenix cells. After 24 h, the culture media containing the virus were filtered through a 0.45-μm syringe filter and added to 60% confluent MS-1 cells with 4 μg/mL of polybrene. For selection of infected cells, the media was changed to growth media (Dulbecco's modified Eagle's medium containing 5% fetal bovine serum and 0.5 μg/mL of puromycin) and maintained for 2 weeks. For TAZ knockdown, pSRP-mTAZ plasmid (31795, Addgene, Watertown, MA, USA) was used.

### Luciferase reporter gene assay

Nos3 gene promoter region, covering two KLF2-binding motifs, was amplified using polymerase chain reaction (PCR) with Phusion™ polymerase (Thermo Scientific). The PCR primer sequences used for amplification of the mouse Nos3 gene promoter were: Forward: 5′-AAAAAAACTCGAGGTGGGTTCAGGAAATTGAGATGA-3′ and reverse: 5′-AAAAAAAAAGCTTAGCAGAGTCCTGGCCTT-3′. The amplified PCR products and the pGL3-basic luciferase vector were digested with XhoI and HindIII, and ligated to generate the pGL3-Nos3 promoter construct. For the luciferase reporter gene assay, HEK293T cells were transfected with the pGL3-Nos3 promoter, along with KLF2- and/or TAZ-expressing vectors. A Renilla luciferase-expressing vector was transfected under all conditions, for normalization. X-tremeGENE™ 9 transfection reagent (Sigma-Aldrich) was used for transfection. Twenty-four hours after transfection, the cells were lysed and luciferase activity was measured using the Dual-Luciferase® Reporter Assay System (Promega, Madison, WI, USA). The value of the firefly luciferase activity for the pGL3-Nos3 promoter was normalized to that of Renilla luciferase. Data have been presented as fold relative to the control condition.

### Immunostaining

Mouse livers were fixed in 4% paraformaldehyde, at 4 °C, for 48 h. The fixed liver was then dehydrated using an ethanol series, soaked in xylene, and embedded in paraffin, at 60 °C overnight. Incubated paraffin was cooled at room temperature and sliced into 5-mm tissue sections using a microtome and attached to a glass slide. The tissues were rehydrated, and antigen retrieval was performed using a pressure cooker. In case of 3,3'-diaminobenzidine tetrahydrochloride (DAB) staining, tissue sections were incubated in 3% H2O2, to block any endogenous peroxidase activity. The tissue sections were then blocked using 1.5% normal goat serum diluted in phosphate-buffered saline (PBS) and an Avidin/Biotin Blocking Kit (SP-2001, Vector Laboratories, Burlingame, CA, USA). The tissue sections were incubated with primary antibodies diluted in 2.5% normal goat serum, at 4 °C overnight. After two washes with PBST (8 mM Na2HPO4, 150 mM NaCl, 2 mM KH2PO4, 3 mM KCl, and 0.05% Tween 20, pH 7.4), fluorophore-conjugated or biotinylated secondary antibodies diluted in PBS were added to the samples, and incubated at room temperature, for 1 h. For immunofluorescence, the tissue sections were washed twice with PBST and mounted with DAPI-containing mounting solution (SP H-1500-10, Vector Laboratories). A confocal microscope (LSM510 META and Axio Observer 7 equipped at Ewha Drug Development Research Core Center; Carl Zeiss, Germany) was used for image acquisition. Zeiss LSM 5 software (v3.2) was used to acquire images, and Zeiss LSM Image Examiner (v4.0.0.241) was used for fluorescence image processing. For immunohistochemistry, the VECTASTAIN® ABC Kit Peroxidase (HRP) (PK-4001, Vector Laboratories) was used. Briefly, samples were washed twice with PBST and incubated with a mixed solution containing avidin and biotinylated horseradish peroxidase (HRP), for 30 min. The samples were then incubated with DAB substrate (SK-4100, Vector Laboratories), counterstained with Mayer's hematoxylin (S3309, Agilent, Santa Clara, CA, USA), and cleared with an ethanol series and xylene. After drying the remaining xylene in the hood, the tissues were mounted using a toluene-based mounting solution (6769007, Thermo Fisher Scientific, Waltham, MA, USA). Sample images were acquired using a light microscope.

### Sirius Red staining

Paraffinized tissue sections were rehydrated and stained with Sirius Red solution (1 mg/mL in picric acid), for 1 h. The stained tissues were rinsed twice with 0.5% acetic acid and dehydrated in 100% ethanol. After clearance with xylene, the specimens were mounted and observed under a light microscope.

### Hematoxylin and eosin (H&E) staining

After sacrifice, the livers were isolated from the mice and fixed with 4% paraformaldehyde, at 4 °C for 48 h. The liver was then dehydrated with an ethanol series, treated with xylene, and embedded in Paraplast®, at 60 °C for 16 h. After incubation, the liver paraffin block was cooled to room temperature and sliced into a 5 μm-thick paraffin ribbon, which was attached to a glass slide. Slides were incubated at 60 °C, for 20 min, and then rehydrated. The slides were incubated in Harris hematoxylin solution for staining, differentiated with 1% acidic alcohol, and incubated in 0.2% of ammonia water. The specimens were counterstained with eosin Y solution, followed by dehydration in an ethanol series. Finally, the samples were cleaned with xylene and mounted with a xylene-based mounting medium. Images of the sections were observed and obtained using a light microscope and an attached digital camera.

### Oil Red O staining

Frozen livers were sectioned to a thickness of 10 μm and mounted on glass slides. Tissues were air dried at room temperature, for 1 h, and then fixed in ice cold 10% formalin, for 10 min. The tissues were then rinsed with distilled water and air-dried for 5 min. Specimens were placed in absolute propylene glycol, for 5 min, and stained with pre-warmed 0.5% Oil Red O solution, at 60 °C for 10 min. The tissues were incubated in 85% propylene glycol, for 5 min, and then rinsed in two changes of distilled water. After counterstaining with Mayer's hematoxylin for 30 s, the specimens were washed thoroughly under running tap water, for 3 min. The stained tissues were mounted in an aqueous mounting medium.

### Scanning electron microscopy

The mice were sacrificed, following which their livers were isolated and transferred to a Petri-dish filled with sterilized PBS (pH 7.4), at 37 °C. PBS was directly injected into the livers, using a 25G syringe. Following that, 1.5% glutaraldehyde was injected into the livers, using a 25G syringe, until tissue hardening was observed. Fixed liver was minced into sections of 1 mm × 1 mm × 1 mm size and incubated in 1.5% glutaraldehyde solution, at 4 °C overnight. After three washes with 0.05 M sodium cacodylate buffer, the tissues were further fixed with 1% osmium tetroxide, for 1 h at 4 °C. After fixation, the tissues were washed three times with distilled water and dehydrated with an ethanol series. The specimens were dried using a critical point dryer, as indicated by the manufacturer's protocol. The dried specimens were mounted on a stub with copper tape and coated with platinum, at a thickness of 15 nm. The platinum-coated samples were observed under a field-emission scanning electron microscope (AURIGA®, Carl Zeiss).

### NO quantification

Mouse sera or culture media were acquired, and NO levels were quantified using a NO colorimetric assay kit (K262, BioVision, Waltham, MA, USA). In case of measurement using cell culture media, the cells were harvested, and the total protein mass was quantified for normalization. Mouse serum was prepared by collecting blood by means of tail bleeding, followed by centrifugation after blood clotting.

### Cyclic guanosine monophosphate (cGMP) quantification

The livers were isolated and immediately frozen in liquid nitrogen. The weight of the isolated liver was measured and 0.1 M HCl was added to the tissue. Liver samples were homogenized using a Polytron™-type homogenizer and centrifuged at 14,000 × g, for 5 min, to collect the supernatant. The supernatant was used directly for the assay. The cGMP Direct Immunoassay Kit (K372-100; BioVision) was used for analysis. Subsequent experimental steps were performed following the manufacturer's protocol.

### Alanine aminotransferase (ALT) activity assay

Mouse serum was prepared by collecting blood by means of tail bleeding, followed by centrifugation after blood clotting, and incubated at room temperature for 30 min. The supernatant was collected and directly assessed using an Alanine Aminotransferase (ALT or SGPT) Activity Colorimetric/Fluorometric Assay Kit (K752-100, BioVision). The subsequent experimental steps were performed according to the manufacturer's protocol.

### Aspartate aminotransferase (AST) activity assay

Mouse serum was prepared by tail bleeding. The isolated serum was assayed directly using an aspartate aminotransferase (AST) activity assay kit (ab105135, Abcam). Subsequent experimental steps were performed according to the manufacturer's protocol.

### Cholesterol quantification

Mouse serum was prepared by tail bleeding and analyzed directly using the cholesterol/cholesteryl ester quantitation assay kit (ab65359, abcam). For liver tissue, 10 mg of isolated liver was used for lipid extraction. Subsequent experimental steps were performed according to the manufacturer's protocol.

### Triglyceride quantification

Mouse serum was prepared by tail bleeding and analyzed directly using triglyceride assay kit (ab65336, abcam). For liver tissue, 100 mg of isolated liver was used for assay. Subsequent experimental steps were performed according to the manufacturer's protocol.

### Isolation of Kupffer cells

Mouse Kupffer cell (KC) were isolated from eight- to ten-week-old mice by the collagenase perfusion method 23. Briefly, collagenase 96 IV-perfused mouse liver was shaken in buffer to dissociate cells. Cell-containing buffer was filtered through a 40 μm Corning® cell strainer (CLS431750, Merck). The non-parenchymal cell (NPC) population was then isolated by centrifugation at 25 × g. The supernatant NPC fraction was separated and pelleted by centrifugation at 300 × g. The NPC 100 pellet was resuspended in culture media and placed on Percoll gradient cusion 101 (50% and 25% Percoll). After centrifugation at 900 × g for 25 min, NPCs were collected at the interface between 50% and 25% Percoll. The cells were then seeded in a 6-well plate at a density of 1-3 × 10^7^/well in RPMI-1640 medium containing 1% of penicillin/streptomycin supplemented with 10% fetal bovine serum and incubated for 2 h in a 5% CO_2_ atmosphere at 37 °C. Non-adherent cells were then removed from the dish by gentle washing with PBS, and the adherent cells were KCs.

### Gene expression analysis

Total RNA was isolated from tissues or cells using TRIzol™ reagent, and cDNA was synthesized using M-MLV reverse transcriptase. Transcripts were analyzed with primers (Table S1) for each gene, using a LightCycler® 480 system. Gapdh was assessed along with each experimental set, to normalize the Ct value of the target gene mRNA. The ratio of target gene expression to reference gene expression was calculated using the following formula: amplification efficiency (Ct reference - Ct target). The ratio data are shown as relative fold to the control.

### Co-immunoprecipitation

HEK293T cells were transfected with a FLAG-tagged TAZ and/or KLF2 expression vector. At 24 h after transfection, the cells were harvested and lysed in radioimmunoprecipitation assay buffer containing a protease inhibitor cocktail. Lysates were treated with an anti-FLAG antibody and incubated at 4 °C, overnight. Following that, protein G beads were added to the samples and incubated at 4 °C, for 6 h. The captured proteins were treated with 2× sodium dodecyl sulfate (SDS) sample buffer and boiled at 95 °C, for 10 min, for elution. The precipitated samples were assessed by means of immunoblotting of total cell lysates. For detection of endogenous interaction, FLAG-tagged TAZ-overexpressing MS-1 cells were immunoprecipitated with an anti-FLAG antibody, as mentioned above.

### ChIP-quantitative PCR (ChIP-qPCR)

For crosslinking, formaldehyde was added to the cells, at a final concentration of 0.75%, followed by the addition of 125 mM glycine, to stop the crosslinking reaction. The cells were washed three times with ice-cold PBS, collected in ice-cold PBS, and subjected to centrifugation. Cell pellets were lysed in FA lysis buffer [50 mM HEPES-KOH (pH 7.5), 140 mM NaCl, 1 mM ethylenediaminetetraacetic acid (EDTA) (pH 8.0), 1% Triton™ X-100, 0.1% sodium deoxycholate, 0.1% SDS, and protease inhibitor cocktail] and sonicated using a Bioruptor sonicator (Diagenode). Chromatin was sheared into 500-1000 bp fragments. Fragmented DNA concentrations were measured, and 20 μg of sheared chromatin was used. FLAG-TAZ-bound DNA fragments were captured by adding anti-FLAG antibodies and protein G beads (P-3296, Sigma-Aldrich) coated with bovine serum albumin and salmon sperm DNA, followed by incubation at 4 °C, for 16 h. After washing three times with wash buffer [0.1% SDS, 1% Triton™ X-100, 2 mM EDTA (pH 8.0), 150 mM NaCl, and 20 mM Tris-HCl (pH 8.0)], the samples were rinsed once with the final wash buffer [0.1% SDS, 1% Triton™ X-100, 2 mM EDTA (pH 8.0), 500 mM NaCl, and 20 mM Tris-HCl (pH 8.0)] and eluted with elution buffer (1% SDS and 100 mM NaHCO3), at 30 °C for 15 min, with gentle rotation. Eluted samples were reverse crosslinked by adding RNase A and incubating at 65 °C, for 5 h. ChIPed DNA samples were purified using a Gel Extraction/PCR Purification Kit (Thermo Fisher Scientific). Purified samples were assessed using qPCR (LC480, Roche, Basel, Switzerland). ChIPed DNA was quantified as a percentage of the input fraction, by using the following formula: amplification efficiency ^ (Ct Input - Ct ChIPed). The acquired values were normalized to those of the IgG control group.

### In silico motif analysis

For motif analysis, approximately 500 bases of the upstream Nos3 gene promoter region was analyzed using the PROMO virtual laboratory program (TRANSFAC v8.3) and JASPAR.

### Statistical analysis of data

For the *in vivo* data, all data are presented as mean ± standard error of the sample number. All *in vitro* data are presented as mean ± standard deviation of at least three independent experimental replicates. The data were assessed for statistical significance using unpaired Student's t-test or one-/two-way analysis of variance, with Tukey's or Sidak's multiple-comparisons tests. The significance level is indicated in the figure, with asterisks, and in the Figure legend, as p-values. Prism 6 (v6.01; GraphPad) was used for statistical analysis.

## Results

### Depletion of endothelial TAZ causes capillarization of LSECs in eKO mice

**Figure 1:**
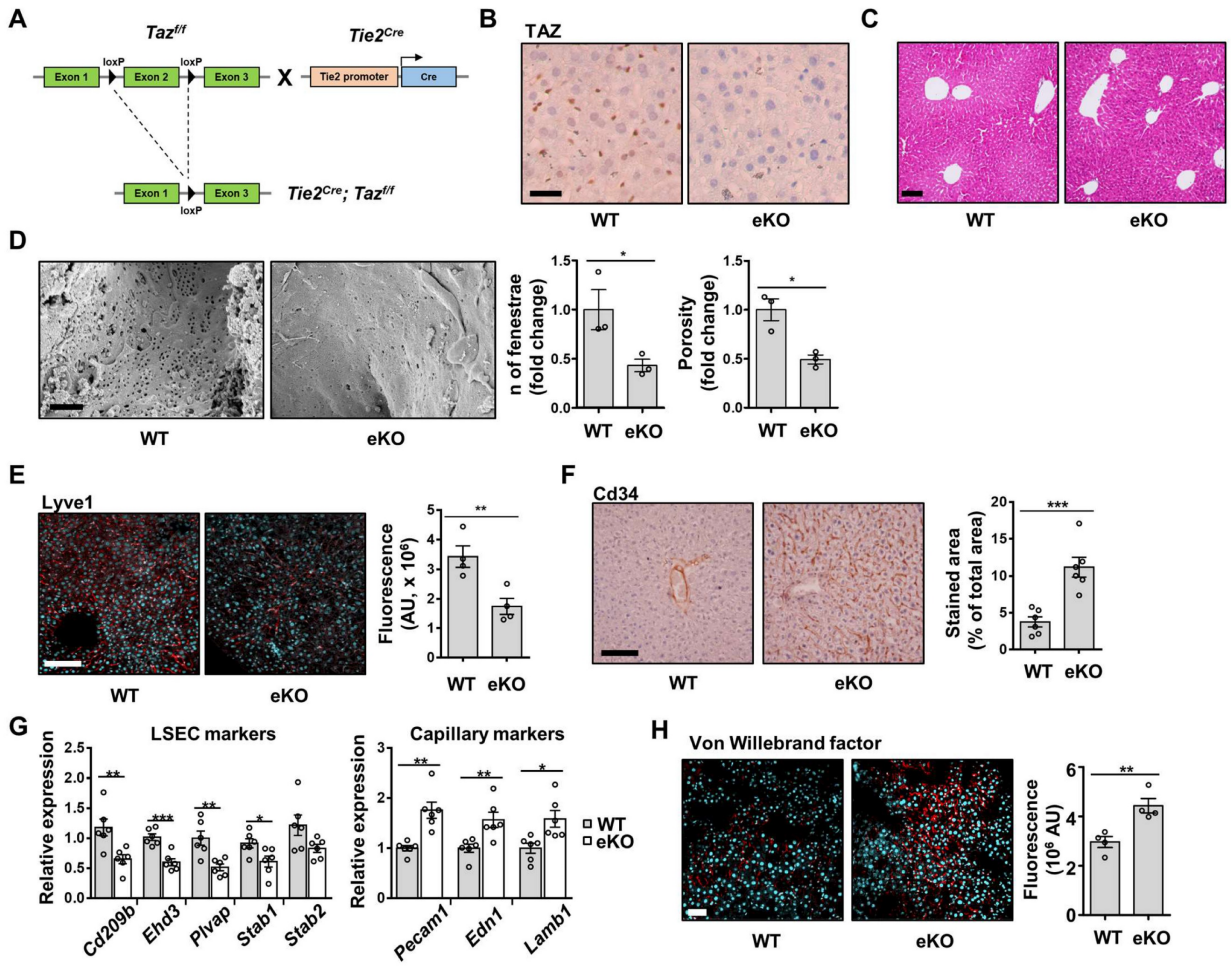
Endothelial TAZ-knockout induces the capillarization of liver sinusoidal endothelial cells. **A)** Strategy for development of endothelial-specific TAZ-knockout (eKO) mice. LoxP-flanked exon 2 region of TAZ was deleted using endothelial-specific cre-recombinase. **B)** Immunostaining of TAZ in wild-type (WT) and eKO mouse livers. Cell nuclei were counterstained with Mayer's hematoxylin. Scale bar = 20 μm. **C)** The histological phenotypes of WT and eKO livers were assessed using H&E staining. Scale bar = 50 μm. **D)** Fenestrae of liver sinusoidal endothelial cells of WT and eKO mice were visualized using a scanning electron microscope. Provided data are representative images from plural mice. Number of fenestrae and porosity was measured and calculated using ImageJ software (n = 3). Scale bar = 1 μm. **E)** WT and eKO mice livers were immunostained with an anti-Lyve1 antibody. 4′,6-diamidino-2-phenylindole (DAPI) was used for nucleus counterstaining. Fluorescence was measured using ImageJ software and presented as an arbitrary unit (n = 4). Scale bar = 100 μm. **F)** WT and eKO mouse livers were immunostained with an anti-Cd34 antibody. DAB signal intensity was assessed and calculated using ImageJ (n = 6). Scale bar = 100 μm. **G)** Expression of the indicated LSEC and capillary marker genes was analyzed using quantitative real-time-polymerase chain reaction (n = 6). **H)** Expression of Von Willebrand factor was assessed using immunofluorescence. The cell nucleus was counterstained with DAPI. The fluorescence signal was measured and calculated using ImageJ (n = 4). Scale bar = 50 μm. Eight to ten-week-old mice were used for all panels. Data are presented as mean ± SEM. (*P < 0.05, **P < 0.01, and ***P < 0.0005, using two-tailed Student's t-test).

To investigate the role of TAZ in endothelial cells, eKO mice were generated by crossing Tie2-cre mice with TAZ-floxed mice (Figure 1A). Endothelial TAZ depletion was confirmed by immunostaining of liver tissue in wild-type (WT) and eKO mice (Figure 1B). On H&E staining, there was no structural alteration of liver tissue in the eKO mice, as compared to that in the WT mice (Figure 1C). Next, scanning electron microscopy was assessed to study the detailed structure of the LSECs. Notably, the number of fenestrae and porosity were significantly decreased in the eKO LSECs (Figure 1D), as compared to that in the WT LSECs. In addition, eKO mouse livers showed decreased levels of Lyve1, which is a sinusoidal endothelial marker (Figure 1E), and increased levels of CD34, a capillary endothelial marker, upon immunostaining (Figure 1F). Similar results were obtained using qRT-PCR. As shown in Figure 1G, the transcription of sinusoidal endothelial markers, including CD209 antigen-like protein B (Cd209b), EH domain-containing protein 3 (Ehd3), plasmalemma vesicle-associated protein (Plvap), stabilin-1 (Stab1), and stabilin-2 (Stab2) were lower in the livers of eKO mice, as compared to those in the livers of the WT mice. However, the transcription levels of capillary endothelial markers, including platelet endothelial cell adhesion molecule (Pecam1), endothelin-1 (Edn1), and laminin subunit beta-1 (Lamb1), were higher in the livers of eKO mice (Figure 1G), as compared to those in the livers of WT mice. Furthermore, the immunostained area for vWF, a marker of liver endothelial dysfunction, was increased in the eKO mice (Figure 1H), as compared to that in the livers of WT mice. As shown in Figure S1, there were no significant differences in endothelial marker gene expression between WT and eKO endothelium of muscle and aortic valve endothelium. Taken together, these results suggested that endothelial TAZ-knockout facilitates capillarization of LSECs.

### Depletion of endothelial TAZ decreases NO levels, through downregulation of eNOS expression in eKO mice

**Figure 2:**
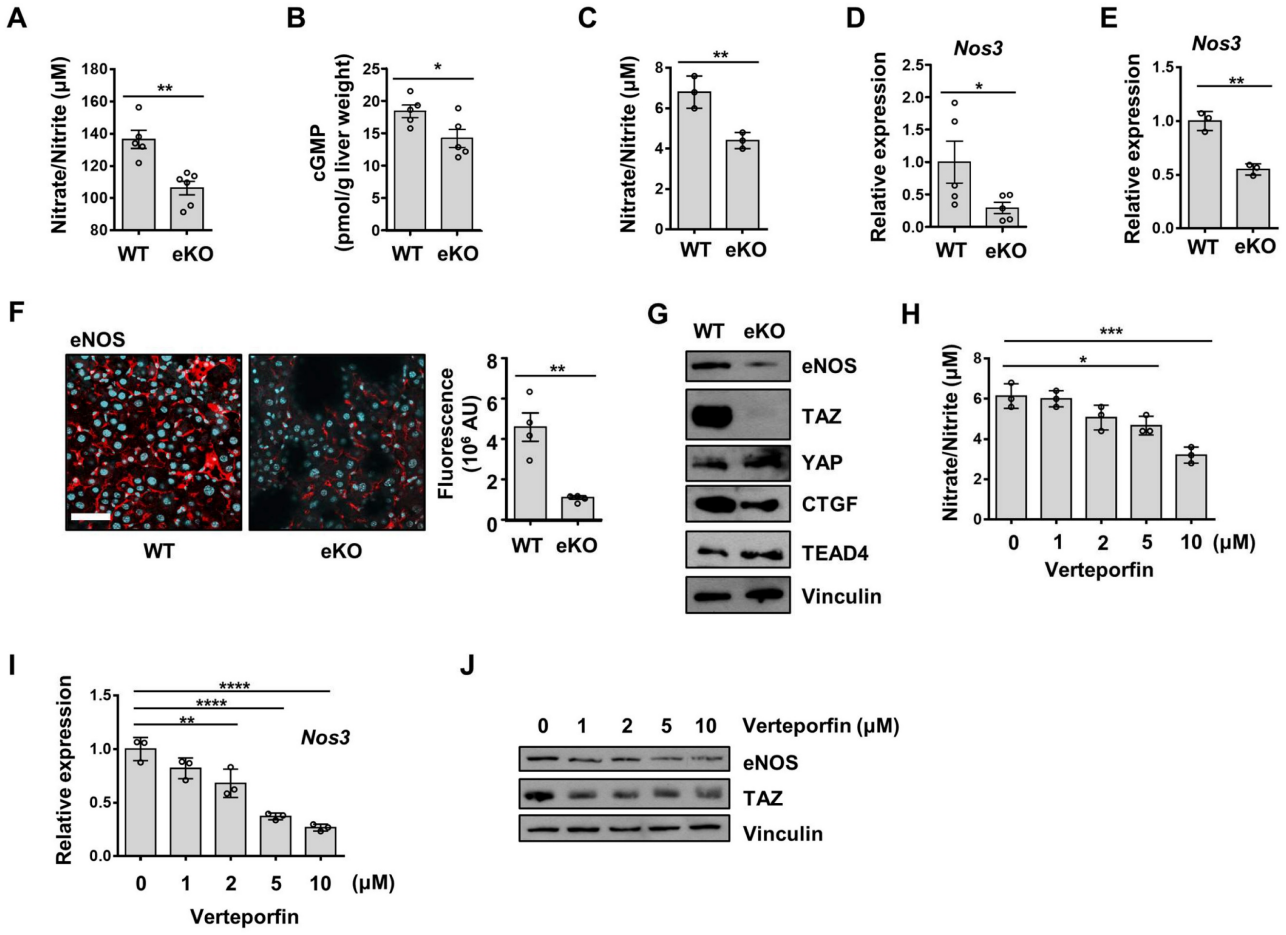
Endothelial TAZ depletion decreases NO production. **A)** Serum was isolated from wild-type (WT) and endothelial-specific TAZ-knockout (eKO) mice, to quantify the nitric oxide (NO) levels. The NO levels were measured using an NO quantification assay kit (n = 5 for WT mice and n = 6 for eKO mice). **B)** Liver homogenates were prepared from WT and eKO mice. cGMP mass was normalized to the wet weight of the used liver (n = 5). **C)** WT and eKO liver sinusoidal endothelial cells (LSECs) were seeded on culture plates. The NO in the culture media was quantified at 24 h after seeding. **D)** Nos3 gene transcription was analyzed using quantitative real-time-polymerase chain reaction (qRT-PCR) in liver of WT and eKO mice (n = 5). **E)** Nos3 gene transcription in the WT and eKO LSECs was assessed using qRT-PCR. **F)** WT and eKO mice livers were immunostained with anti-eNOS antibodies (red). Nuclei were counterstained with 4′,6-diamidino-2-phenylindole (DAPI, blue). The presented data are representative images of multiple mice. The stained area was measured and calculated using ImageJ software (n = 4). Scale bar = 50 μm. **G)** Levels of indicated proteins in the WT and eKO LSECs were analyzed using immunoblotting. Vinculin was used as a loading control. **H)** LSECs were treated with verteporfin at the indicated concentration, for 24 h. The NO levels in the culture media were quantified using a NO quantification assay kit. **I**) The Nos3 transcription levels in panel **H** were analyzed using qRT-PCR. **J)** eNOS proteins in panel **H** were analyzed using immunoblot assay. The experiment was carried out in triplicate (**C**, **E**, **H**, and **I**). Eight to ten-week-old mice were used for all panels. Data are presented as mean ± SEM (**A**, **B**, **D**, and **F**) or mean ± SD (**C**, **E**, **H**, and **I**). (*P < 0.05, **P < 0.01, ***P < 0.0005, and **** P < 0.0001, **A**-**C**; as assessed using two-tailed Student's t-test, **D**-**F**; as assessed using one-tailed Student's t-test, **H**-**I;** as assessed using one-way ANOVA with Tukey's multiple-comparison test).

NO generated by endothelial cells diffuses into smooth muscle cells and interacts with the heme group of soluble guanylate cyclase (sGC), to produce cGMP from guanosine triphosphate. cGMP induces vasodilation of smooth muscle cells 24. Reduced NO bioavailability in LSECs is known to induce defenestration and endothelial dysfunction 10. To investigate NO bioavailability in the livers of WT and eKO mice, we analyzed NO levels, and observed decreased serum NO levels in the eKO mice, compared to those in the WT mice (Figure 2A). cGMP levels were also downregulated in the liver of the eKO mice (Figure 2B), as compared to those in the livers of the WT mice. Similarly, decreased NO levels were observed in the culture media of eKO LSEC, as compared those in the culture media of WT LSEC (Figure 2C). NOS catalyzes the production of NO from L-arginine. eNOS is an isoform of NOS that is constitutively expressed in LSECs 25. We assumed that the decreased NO level in eKO mice was due to the TAZ-mediated transcriptional regulation of eNOS expression. To test this hypothesis, Nos3 mRNA levels in WT and eKO livers were analyzed using qRT-PCR. As shown in Figure 2D, Nos3 transcript levels were downregulated in the eKO mice. Similar results were observed in eKO LSEC (Figure 2E). In addition, decreased eNOS protein levels were observed in the eKO mice, as compared to those in the WT mice, upon immunostaining (Figure 2F). In addition, eKO LSEC showed decreased eNOS protein levels, as determined using immunoblot assay (Figure 2G). The expression of TAZ target genes such as Ctgf and Cyr61 was reduced in eKO mice compared to WT mice (Figure S2). Similarly, Ctgf protein levels were also reduced in eKO mice. YAP and TEAD4 protein levels were not altered in WT and eKO mice (Figure 2G). To further verify the source of NO, the expression of eNOS and iNOS was analyzed in Kupffer cells. As shown in Figure S3, the levels of iNOS and eNOS in Kupffer cells were not altered in WT and eKO mice. Furthermore, upon administration of verteporfin, an inhibitor of YAP/TAZ function 26, to LSECs, the pharmacological inhibition of TAZ also decreased NO production in a dose-dependent manner (Figure 2H). In addition, downregulation of Nos3 transcription (Figure 2I) and eNOS protein levels (Figure 2J) was observed after verteporfin treatment. Thus, our results suggested that TAZ stimulates NO production through induction of eNOS protein levels.

### Rescue of NO signal restores TAZ depletion-induced endothelial dysfunction

**Figure 3:**
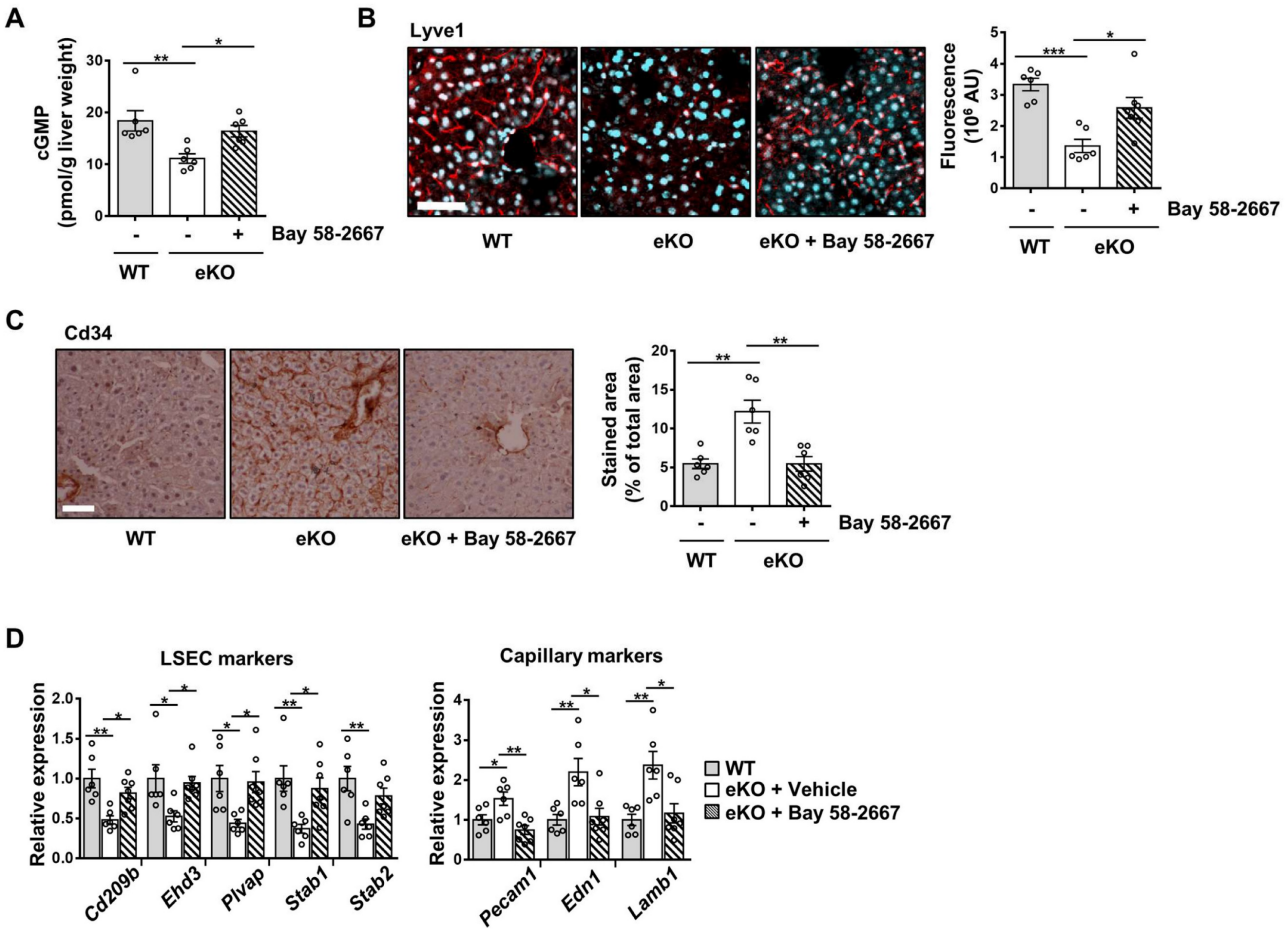
Rescued nitric oxide signaling restores the levels of sinusoidal endothelial markers in endothelial TAZ-KO mice. **A)** cGMP levels were quantified in the liver homogenates of wild-type (WT) mice and vehicle- or sGC activator (BAY 58-2667)-administered endothelial TAZ-knockout (eKO) mice. Data were normalized to the used liver weight (n = 6). **B)** Liver tissues in panel **A** were immunostained using anti-Lyve1 antibodies (red). The cell nuclei were counterstained with 4′,6-diamidino-2-phenylindole (DAPI, cyan). Fluorescence was quantified using ImageJ software and presented as arbitrary units (AUs) (n = 6). Scale bar = 50 μm. **C)** The liver tissues in panel **A** were immunostained with anti-Cd34 antibodies. The stained area was calculated using ImageJ (n = 6). Scale bar = 100 μm. **D)** Mouse liver transcripts in panel **A** were analyzed using quantitative real-time-polymerase chain reaction, to quantify transcripts of the indicated LSEC and capillary marker genes (n = 6). Eight to ten-week-old mice were used for all panels. Data are presented as mean ± SEM. (*P < 0.05, **P < 0.01, and ***P < 0.0005, as assessed using one-way ANOVA with Tukey's multiple-comparison test).

**Figure 4:**
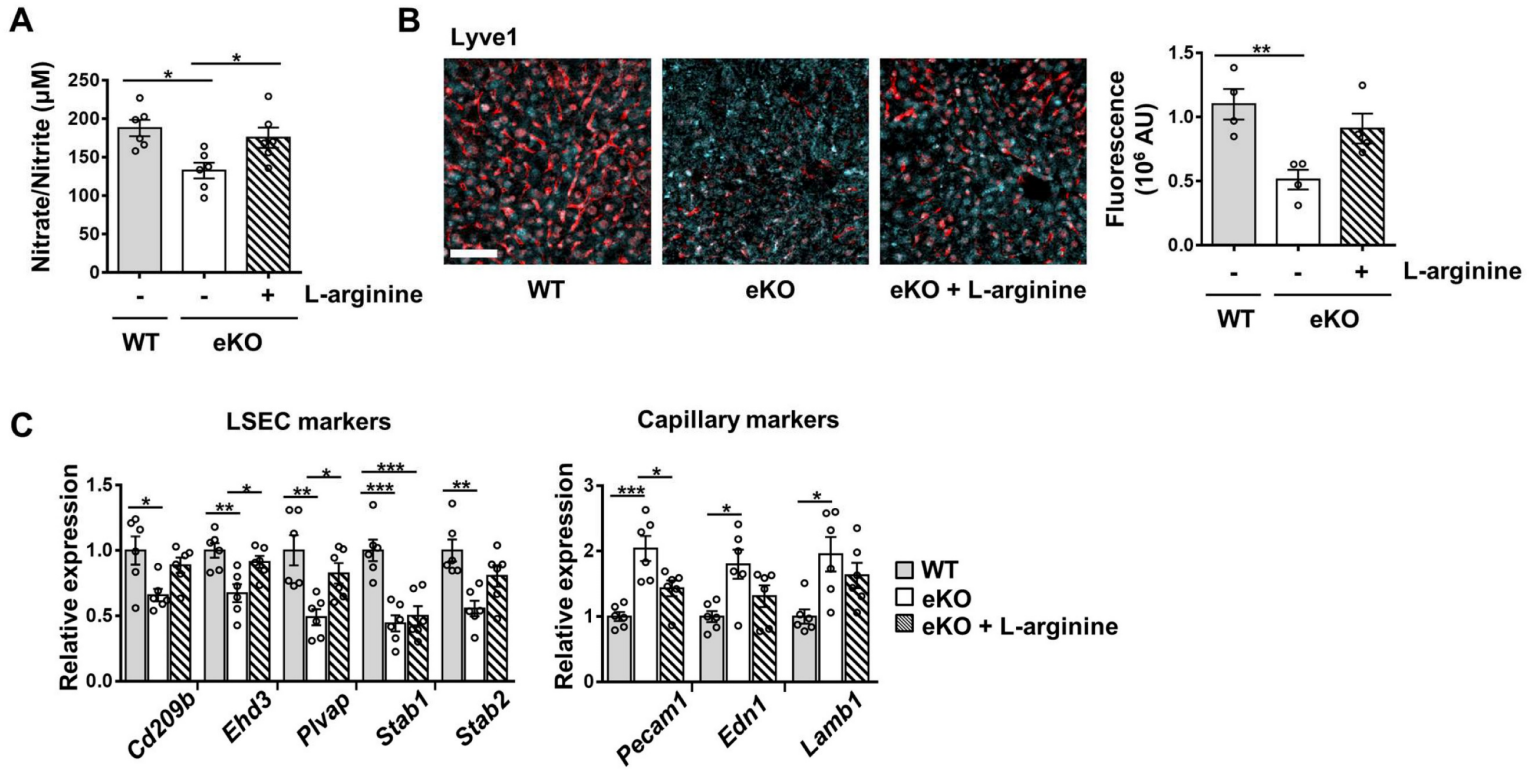
Addition of nitric oxide (NO) restores the level of sinusoidal endothelial markers in endothelial TAZ-KO mice. **A)** NO levels were analyzed in the serum of wild-type (WT) and endothelial TAZ-knockout (eKO) mice provided or not provided with 25 g/L of L-arginine (n = 6). **B)** Liver tissues in panel **A** were immunostained with anti-Lyve1 antibodies, and the fluorescence was quantified using ImageJ (n = 4). Scale bar = 50 μm. **C)** Gene expression of LSEC and capillary markers in panel **A** was assessed using quantitative real-time-polymerase chain reaction (n = 6). Eight to ten-week-old mice were used for all panels. Data are presented as mean ± SEM. (*P < 0.05, **P < 0.01, and ***P < 0.0005, as assessed using one-way ANOVA with Tukey's multiple-comparison test).

To further study the NO signaling pathway in TAZ-mediated LSECs, vehicle or BAY 58-2667, an sGC activator, was administered to eKO mice. As shown in Figure 3A, increased cGMP levels were observed in BAY 58-2667-administered eKO mice, indicating a restored NO signal (Figure 3A). In addition, in BAY 58-2667-administered eKO mice, there was a partial restoration of Lyve1 level, as evidenced by immunofluorescence staining (Figure 3B). However, Cd34 protein levels decreased after administration of BAY 58-2667 (Figure 3C). Finally, administration of BAY 58-2667 restored the transcription of sinusoidal endothelial markers (Cd209b, Ehd3, Plvap, Stab1, and Stab2), but decreased the transcription of capillary endothelial markers (Pecam1, Edn1, and Lamb1) (Figure 3D). Next, we supplied L-arginine, a substrate of eNOS, to upregulate the NO concentration in the livers of eKO mice. As shown in Figure 4A, L-arginine-administered eKO mice displayed restored NO concentration in the serum. In addition, Lyve1 levels were partially restored in the L-arginine-treated eKO mice (Figure 4B). In addition, L-arginine administration partially restored the transcription of sinusoidal endothelial markers (Cd209b, Ehd3, Plvp1, Stab1, and Stab2), but decreased the transcription of capillary endothelial markers (Pecam1, Edn1, and Lamb1) (Figure 4C). Thus, these results suggested that TAZ plays an important role in maintaining sinusoidal endothelial structures, *via* NO-sGC signaling.

### TAZ stimulates Nos3 transcription with the KLF2 transcription factor

**Figure 5:**
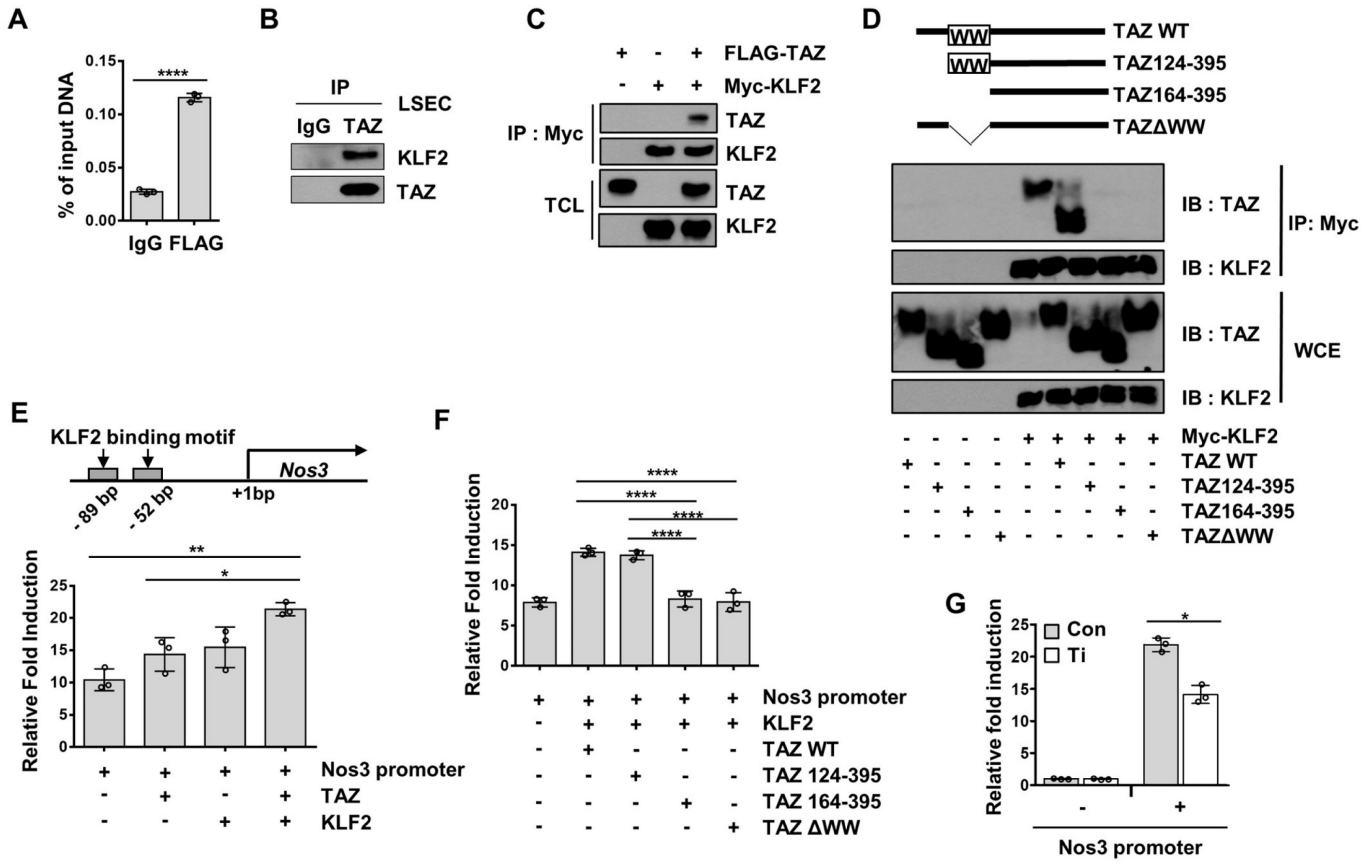
TAZ regulates Nos3 gene transcription with the KLF2 transcription factor. **A)** Liver sinusoidal endothelial cells (LSECs) were assessed using chromatin immunoprecipitation-quantitative polymerase chain reaction, to evaluate the recruitment of TAZ on the Nos3 promoter region. **B)** LSECs were immunoprecipitated with anti-TAZ antibodies and the interaction between TAZ and KLF2 was confirmed using immunoblot assay. IgG was used as an immunoprecipitation control. **C)** Physical interaction of FLAG-tagged TAZ and Myc-tagged KLF2 was verified in HEK293T cells, through a co-immunoprecipitation assay. **D)** Wild-type (WT) or deletion mutants of TAZ plasmids were introduced into HEK293T cells, along with a Myc-tagged KLF2 expression plasmid. Interaction of TAZ and KLF2 was confirmed using immunoprecipitation with Myc-tag antibodies. WCE, whole cell extracts. **E**) Transcriptional activity of the Nos3 promoter-containing luciferase reporter construct was analyzed using a reporter gene assay. The constructed vector was introduced into HEK293T cells, along with the TAZ- and/or KLF2-expressing plasmid. pRL-null renilla luciferase plasmid was used for normalization. **F**) TAZ-WT or -deletion mutant plasmids were transfected into HEK293T cells, along with pGL3-Nos3 promoter and Myc-KLF2 plasmids. Transcriptional activity was assessed *via* a luciferase reporter gene assay. **G**) Control (Con) and TAZ-knockdown (Ti) MS-1 cells were transfected with the pGL3-Nos3 promoter, along with pRL-null renilla luciferase plasmid. Transcriptional activity was assessed using a luciferase reporter gene assay. The experiment was done in triplicate (**A**, **E**, **F**, and **G**). Eight to ten-week-old mice were used for panel **A** and **B**. Data are shown as mean ± SD (*P < 0.05, **P < 0.01, ***P < 0.0005, and ****P < 0.0001, **A**; as assessed using two-tailed Student's t-test, **E** and **F**; as assessed using one-way ANOVA with Tukey's multiple-comparison test, **G**; as assessed using two-way ANOVA with Sidak's multiple-comparison test).

To understand the mechanism of TAZ-mediated transcriptional regulation of Nos3, we searched the transcription factor-binding sites of the Nos3 gene promoter, to identify possible transcription factors that may interact with TAZ. Two KLF2-binding consensus sequences were found around the proximal region of the Nos3 gene promoter. It has been shown that KLF2 induces the transcription of Nos3 in human umbilical vein endothelial cells 27. In addition, the WW domain of TAZ has been shown to interact with the Pro-Pro-X-Tyr amino acid (PPXY) motif of transcription factors 28, and KLF2 has a PPXY motif. Thus, recruitment of TAZ to the Nos3 promoter region covering the KLF2-binding sites was analyzed and confirmed using a ChIP assay (Figure 5A). Next, the physical interactions between TAZ and KLF2 were investigated. As shown in Figure 5B, isolated LSECs were immunoprecipitated using anti-TAZ antibodies, and the interaction between endogenous TAZ and KLF2 was confirmed using immunoblot assay. As shown in Figure 5C, the physical interaction between TAZ and KLF2 was further verified using a co-immunoprecipitation assay, in FLAG-tagged TAZ- and Myc-tagged KLF2-overexpressing HEK293T cells. To determine whether the TAZ WW domain is important for binding to KLF2, we used TAZ-deletion mutants for the co-immunoprecipitation assay. TAZ WT and deletion mutants were introduced into HEK293T cells, along with Myc-tagged KLF2 expression plasmids. As shown in Figure 5D, TAZ WW domain-truncated TAZ mutants, including TAZ164-395 and TAZ∆WW, showed no interaction with Myc-tagged KLF2, when assessed using an immunoprecipitation assay. Next, to evaluate the transcriptional activity of the TAZ-binding region of the Nos3 gene promoter, we cloned the Nos3 gene promoter harboring approximately 500 bp near the transcription start site into a luciferase reporter plasmid, which was transfected into cells with TAZ and/or KLF2 expression plasmids. As shown in Figure 5E, there was an increase in luciferase activity upon TAZ or KLF2 transfection, and a further increase upon introduction of both TAZ and KLF2. However, TAZ deletion in the WW domain did not result in KLF2-induced increased luciferase activity (Figure 5F). Finally, small hairpin RNA-driven TAZ knockdown decreased the luciferase activity, as compared to that in the control (Figure 5G). Thus, these results suggested that TAZ stimulates KLF2-induced Nos3 transcription.

### Endothelial-specific TAZ-knockout stimulates liver fibrosis after liver damage

**Figure 6:**
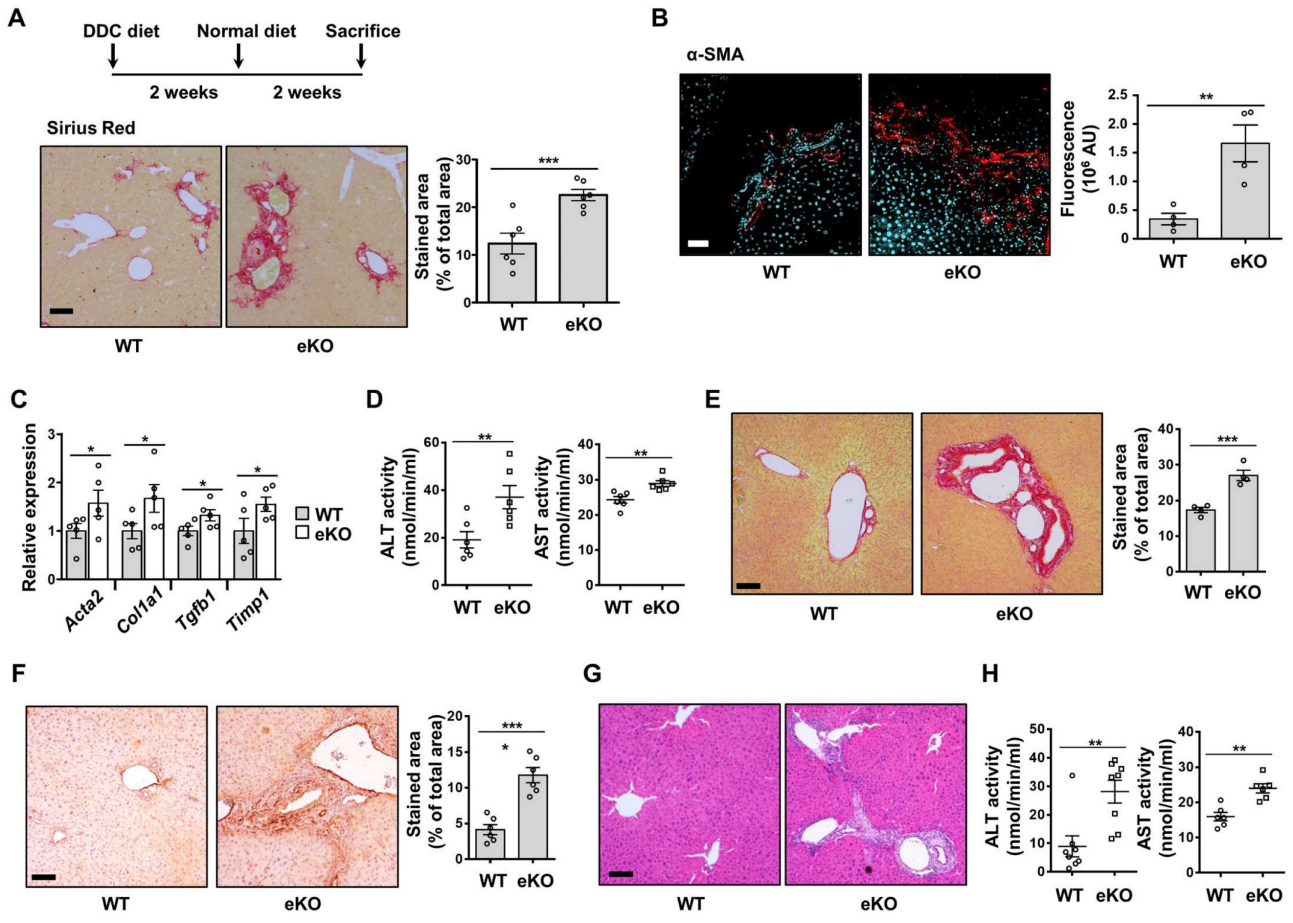
DDC diet-induced liver fibrosis deteriorates in endothelial TAZ-knockout mice. **A)** Wild-type (WT) and endothelial TAZ-knockout (eKO) mice were fed with a 3,5-diethoxycarbonyl-1,4-dihydrocollidine (DDC) diet, following which their livers were analyzed using Sirius Red staining, to evaluate the degree of fibrosis. The presented data are representative images from multiple mice. The stained area was measured and calculated using ImageJ software (n = 6). Scale bar = 100 μm. **B)** The liver in panel **A** was immunostained using anti-alpha smooth muscle actin (α-SMA) antibodies. The stained area was measured and calculated using ImageJ software (n = 6). Representative images have been shown from multiple mice. Scale bar = 100 μm. **C)** Total RNA was isolated from livers in panel **A** and transcription levels of fibrosis marker genes were assessed using quantitative reverse transcription-polymerase chain reaction. Target gene transcription was normalized to that of *Gapdh*. **D)** Serum isolated from WT and eKO mice was analyzed to determine alanine aminotransferase (ALT) and aspartate aminotransferase (AST) activity (n = 6). **E)** Partial hepatectomy (PHx) was conducted in WT and eKO mice, following which the livers were subjected to Sirius Red staining, at 8 d after PHx. Provided data are representative images from multiple mice. The stained area was measured and analyzed using ImageJ (n = 4). Scale bar = 100 μm. **F)** Livers in panel **E** were immunostained using α-SMA antibodies. The stained area was measured and analyzed using ImageJ (n = 6). Scale bar = 100 μm. **G)** Livers in panel **E** were subjected to H&E staining. Scale bar = 100 μm. **H)** Serum of mice in panel **E** was analyzed to determine the alanine aminotransferase (ALT) and aspartate aminotransferase (AST) activity (n = 8 for ALT and n = 6 for AST). Eight to ten-week-old mice were used for all panels. Data are presented as mean ± SEM. (*P < 0.05, **P < 0.01, and ***P < 0.0005, as assessed using a one-tailed Student's t-test).

To evaluate the role of endothelial TAZ in liver fibrosis after liver damage, WT and eKO mice were fed a DDC diet for two weeks, followed by a normal chow diet for two weeks (Figure 6A). The mice were then sacrificed, and their livers were analyzed using Sirius Red staining, to observe the progression of fibrosis. As shown in Figure 6A, eKO mouse livers showed increased Sirius Red-stained area, as compared to that in the WT mouse livers. Augmented fibrosis in the eKO mouse livers was also verified by α-SMA immunostaining (Figure 6B), and the expression of fibrosis marker genes, including actin alpha 2, smooth muscle, aorta (Acta2), collagen alpha-1(I) chain (Col1a1), transforming growth factor beta-1 (Tgfb1), and metalloproteinase inhibitor 1 (Timp1) (Figure 6C). Damaged livers of eKO mice were further confirmed by observing the increased levels of alanine aminotransferase (ALT) and aspartate aminotransferase (AST) activity (Figure 6D).

To further study the role of endothelial TAZ in liver damage and recovery, two-thirds PHx was performed in the WT and eKO mice. After 8 d, the livers were isolated from the sacrificed mice, to observe for phenotypic differences between the WT and eKO livers. Liver fibrosis was severe in the livers of eKO mice, at 8 d after PHx, as evidenced by Sirius Red staining (Figure 6E) and α-SMA immunostaining (Figure 6F). Notably, the features of primary sclerosing cholangitis (PSC) were also evidenced by the unique periductular onion-skin pattern in the eKO liver, after PHx (Figure 6G). Finally, disrupted liver function was confirmed using ALT and AST activity assay (Figure 6H). Thus, the results showed that loss of endothelial TAZ stimulates liver fibrosis during the recovery from liver damage, suggesting that endothelial TAZ resists damage-induced liver fibrosis.

### Fat accumulation and liver fibrosis are aggravated in the eKO mice, after liver damage

**Figure 7:**
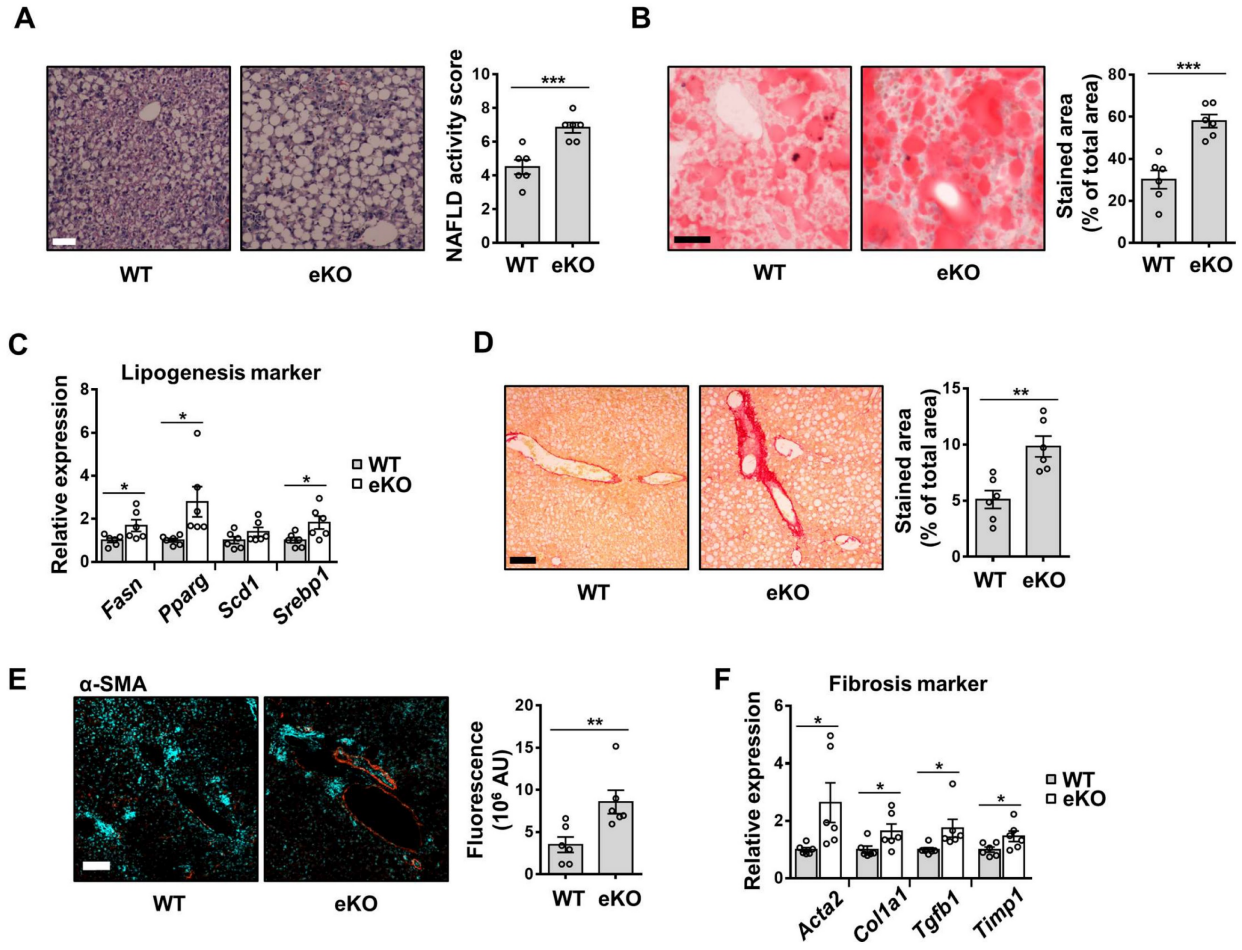
MCD diet-induced NAFLD and NASH is aggravated in endothelial TAZ-knockout mouse. **A)** Wild-type (WT) and endothelial TAZ-knockout (eKO) mice were fed with a methionine-choline-deficient (MCD) diet, for 4 weeks. WT and eKO mice livers were analyzed using H&E staining. The NAFLD activity score was calculated in terms of steatosis area, number of lobular inflammation foci, and degree of hepatocyte ballooning (n = 6). Scale bar = 50 μm. **B)** The liver in panel **A** was cryosectioned, following which the lipid droplet was subjected to Oil Red O staining. The fraction of stained area was measured using ImageJ (n = 6). Scale bar = 50 μm. **C)** Transcripts of mouse livers in panel **A** were analyzed using quantitative real-time-polymerase chain reaction (qRT-PCR), to quantify the gene expression of lipogenesis markers (*Fasn*, *Pparg*, *Scd1*, and *Srebp1*) (n = 6). **D)** To visualize collagen accumulation, mouse livers in panel **A** were subjected to Sirius Red staining. The stained area was measured and analyzed using ImageJ (n = 6). Scale bar = 100 μm. **E)** The liver in panel **A** was stained for alpha smooth muscle actin (α-SMA). The stained area was calculated using ImageJ (n = 6). Scale bar = 100 μm. **F)** The transcripts of mouse livers in panel **A** were measured and analyzed using qRT-PCR, to quantify the gene transcription of fibrosis markers (*Acta2*, *Col1a1*, *Tgfb1*, and *Timp1*) (n = 6). Eight to ten-week-old mice were used for all panels. Data are presented as mean ± SEM. (*P < 0.05, **P < 0.01, and ***P < 0.0005, as assessed using a one-tailed Student's t-test).

Non-alcoholic fatty liver disease (NAFLD) is a major cause of chronic liver disease. It ranges from mild liver steatosis to non-alcoholic steatohepatitis (NASH), which progresses further to cirrhosis 29. The involvement of eNOS in MCD diet-induced NASH development has also been reported 30. To evaluate the effect of endothelial TAZ in MCD diet-induced NASH, WT and eKO mice were fed an MCD diet for four weeks. As shown in Figure 7A and 7B, H&E and Oil Red O staining showed that liver steatosis was more severe in eKO livers than in WT livers. The transcription of lipogenesis marker genes, including fatty acid synthase (Fasn), peroxisome proliferator-activated receptor gamma (Pparg), stearoyl-CoA desaturase 1 (Scd1), and sterol regulatory element-binding protein 1 (Srebp1), was also higher in eKO livers than in WT livers (Figure 7C). In addition, serum and tissue triglyceride and cholesterol levels were analyzed and slightly elevated triglyceride and cholesterol levels were observed in eKO mice compared to WT mice (Figure S4). Fibrosis progression was also accelerated in eKO livers, as evidenced by increased collagen deposition (Figure 7D), α-SMA protein levels (Figure 7E), and fibrosis marker gene levels (Acta2, Col1a1, Tgfb1, and Timp1) (Figure 7F). Thus, these results suggested that the loss of endothelial TAZ aggravates MCD diet-induced lipogenesis and fibrosis.

Taken together, endothelial TAZ plays an important role in maintaining the sinusoidal endothelial structure, through NO production, and resisting liver damage-induced fat accumulation and fibrosis, thereby suggesting an unappreciated role for endothelial TAZ in vascular health and liver disease.

## Discussion

LSECs offer a permeable barrier due to their highly specialized structures, such as fenestrae, and the absence of basement membranes. Defenestration of the liver sinusoidal endothelium is an initial event in the disease, followed by liver fibrosis and cirrhosis. Dysregulation of LSEC function is accompanied by both acute and chronic liver injury, including ischemia-reperfusion injury, hepatic sinusoidal obstruction syndrome, NASH, and NAFLD 2. Our study showed that endothelial TAZ-KO facilitates defenestration of the liver sinusoidal endothelium (Figure 1). Capillarization impedes the normal exchange of chylomicron remnants, thereby potentiating steatosis development 2. Indeed, the pathological importance of endothelial TAZ has been observed in an MCD diet-induced NASH mouse model. After the MCD diet, the eKO mice showed increased lipogenesis and fibrosis, as compared to the WT mice (Figure 7). Our results suggested that endothelial TAZ plays an important role in maintaining the sinusoidal structure of endothelial cells.

Reduction of NO bioavailability is associated with liver disease progression, which induces hepatic vascular resistance, activation of HSCs, and defenestration of LSECs 1. HSCs are quiescent under normal physiological conditions; however, their activation leads to liver fibrosis 4. HSC quiescence is maintained by the NO produced by LSECs. eNOS3 produces NO from L-arginine. In this study, we demonstrated that endothelial TAZ promotes NO production (Figure 2), *via* the induction of eNOS transcription (Figure 5), and suggested that decreased NO production in eKO mice induces HSC activation and defenestration. Thus, loss of TAZ in LSECs may constitute a hepatic microenvironment that favors fibrosis progression in the injured liver.

PHx evokes elevation of fluidal shear stress in liver sinusoids, because the portal flow per gram of tissue is increased 31. Increased shear stress on LSECs promotes NO production, which induces vasodilation 32 and sensitizes hepatocytes to hepatocyte growth factor, for liver regeneration 1. TAZ functions as a mediator of shear stress-induced mechanotransduction 19. Also, it has been shown that YAP/TAZ is involved in angiogenesis, by means of a shear flow-induced atheroprotective effect 18. Therefore, our results suggest that the decreased NO mediated by endothelial TAZ-knockout interrupts the normal regeneration process after PHx, and can induce aberrant alterations in cellular signaling, resulting in the onset of liver fibrosis.

PSC is a cholestatic hepatobiliary disease that is characterized by hepatic fibrosis, chronic inflammation, and edema surrounding interlobular bile ducts 33. The pathological mechanism of PSC is unknown, and there is no effective treatment for it except for liver transplantation. Notably, as described in Figure 6G, we observed a typical onion-skin-like pattern in the portal triad region of the eKO liver, after PHx and a DDC diet (Figure 6G). Defenestration in eKO sinusoids can impede the normal exchange of biomolecules between the liver parenchyma and the circulation, immune cell recruitment, and regeneration. It is speculated that these aberrant responses of TAZ KO in LSECs play a role in the development of the PSC-like phenotype observed in eKO livers, after liver damage. These studies provide new insights into PSC development at the cellular and molecular levels, and novel pharmacological targets for the treatment of PSC and other diseases related to liver fibrosis.

Taken together, endothelial TAZ plays an important role in the maintenance of liver sinusoidal structure, through induction of NO, and protects against liver fibrosis after liver damage caused by a DDC or MCD diet.

## Supplementary Material

Supplementary figures and table.

## Abbreviations

NO: nitric oxide
LSEC: liver sinusoidal endothelial cell
HSC: hepatic stellate cell
TAZ: transcriptional co-activator with PDZ-binding motif
YAP: Yes-associated protein
DDC: 3,5-diethoxycarboncyl-1,4-dihydrocollidine
MCD: methionine-choline-deficient
NOS: nitric oxide synthase
vWF: von Willebrand factor
sGC: soluble guanylate cyclase
KLF2: Krüppel-like factor 2
α-SMA: alpha-smooth muscle actin
Acta2: actin alpha 2, smooth muscle, aorta
Col1a1: collagen alpha-1(I) chain
Tgfb1: transforming growth factor beta-1
Timp1: metalloproteinase inhibitor 1
ALT: alanine aminotransferase
PHx: partial hepatectomy
NAFLD: non-alcoholic fatty liver disease
NASH: non-alcoholic steatohepatitis
