# Supplementary material for: Endothelial TAZ inhibits capillarization of liver sinusoidal endothelium and damage-induced liver fibrosis via nitric oxide production
Source: Theranostics. 2023 Jul 16;13(12):4182–96. doi: 10.7150/thno.83714 (PMC10405847; doi:10.7150/thno.83714)
Supplement: Supplementary file 1 — Supplementary figures and table. [file thnov13p4182s1.pdf]

Supplementary Figures and Table

Endothelial TAZ inhibits capillarization of liver sinusoidal endothelium and damage-induced liver fibrosis via nitric oxide production.

Jun-Ha Hwang[1], Woong Heo[1], Jung Il Park[1], Kyung Min Kim[1], Ho Taek Oh[1], Gi Don Yoo[1], Jeekeon Park[1], Somin Shin[1], Youjin Do[1], Mi Gyeong Jeong[2], Eun Sook Hwang[2]*, Jeong-Ho Hong[1]*

[1]Division of Life Sciences, Korea University, Seoul 02841, Korea

[2] College of Pharmacy and Graduate School of Pharmaceutical Sciences, Ewha Womans University, Seoul 03760, Korea

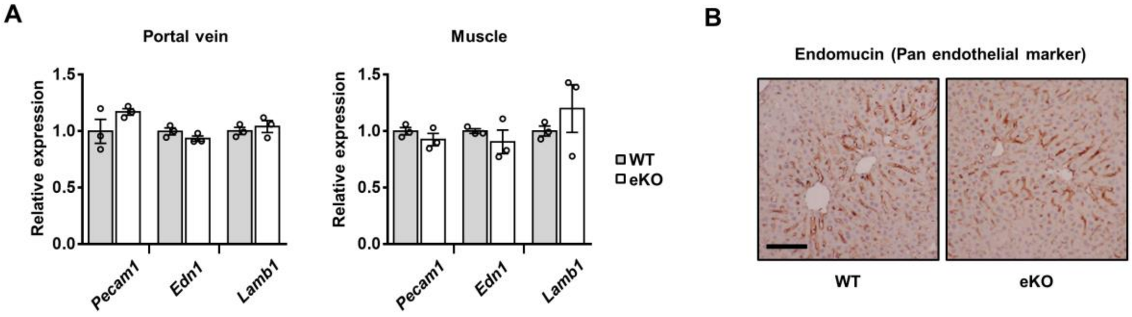

Figure S1. A) Descending aorta and tibialis anterior muscle were isolated from wild-type (WT) and endothelial TAZ-knockout (eKO) mice. After RNA extraction and cDNA synthesis, endothelial marker gene expression was assessed by qRT-PCR (n = 3). **B)** Liver endothelium of WT and eKO mice was visualized by immunostaining for the pan-endothelial marker, endomucin. Scale bar = 200 μm. For panel **A**, data are presented as mean ± SD.

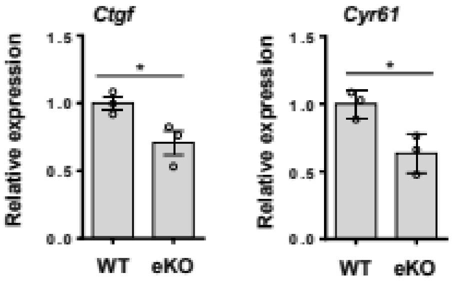

Figure S2. Liver sinusoidal endothelial cells were isolated from wild-type (WT) and endothelial TAZ-knockout (eKO) mice. The relative expression of TAZ target genes was analyzed by quantitative real-time PCR (n = 3). Data are presented as mean ± SD. (*P<0.05, as assessed using a one-tailed Student's t-test).

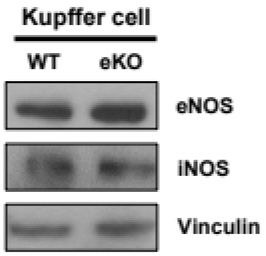

Figure S3. eNOS and iNOS levels in Kupffer cells. Kupffer cells were isolated from WT or eKO mice. The expression of eNOS, iNOS, and vinculin was analyzed by immunoblotting. Vinculin was used as a loading control.

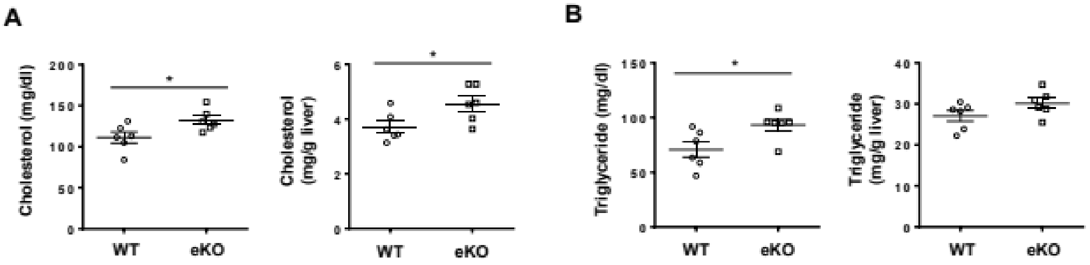

Figure S4. A) Wild-type (WT) and endothelial TAZ-knockout (eKO) mice were fed with a 3,5-diethoxycarbonyl-1,4-dihydrocollidine (DDC) diet. Serum and liver tissue cholesterol levels were quantified using a cholesterol quantification kit (n = 6). **B)** Serum and liver tissue triglyceride levels of the mice in panel **A** were quantified using a triglyceride assay kit (n = 6). Data are presented as mean ± SEM. (*P<0.05, as assessed using a one-tailed Student's t-test).

Table S1

Primers used for gene expression analysis		
Gene	Direction	Sequence (5' → 3')
Cd209b	Forward	TGGGCTCCTGCTGATCATT
	Reverse	TTCCCTTGGGAGATGGGGAT
Ehd3	Forward	CGCCGTGCTTGAAAGTATCAG
	Reverse	ATAATTCGGTCCACCCGCTC
Plvp1	Forward	AGCACACTGCCTTCTCCTTG
	Reverse	AGCACACTGCCTTCTCCTTG
Stab1	Forward	TCACTGTCCCCACACTACTTT
	Reverse	TGTCGCAACGTTTAGACCGTA
Stab2	Forward	CACTATGTCGGGGATGGACG
	Reverse	GGGAGCGTAGGTGGAATACG
Pecam1	Forward	ACCGGGTGCTGTTCTATAAGG
	Reverse	TCACCTCGTACTCAATCGTGG
Edn1	Forward	CACCGTCCTCTTCGTTTTGC
	Reverse	GGCTCTGCACTCCATTCTCA
Lamb1	Forward	GAAAGGAAGACCCGAAGAAAA
	Reverse	CCATAGGGCTAGGACACCAAA
Nos3	Forward	CCTTCCGCTACCAGCCAG
	Reverse	CAGAGATCTTCACTGCATTGGCT
Acta2	Forward	GTTCAGTGGTGCCTCTGTCA
	Reverse	ACTGGGACGACATGGAAAAG
Col1a1	Forward	TAGGCCATTGTGTATGCAGC
	Reverse	ACATGTTCAGCTTTGTGGACC
Tgfb1	Forward	GTGGAAATCAACGGGATCAG
	Reverse	ACTTCCAACCCAGGTCCTTC
Timp1	Forward	AGGTGGTCTCGTTGATTTCT
	Reverse	GTAAGGCCTGTAGCTGTGCC
Fasn	Forward	AGGGGTCGACCTGGTCCTCA
	Reverse	GCCATGCCCAGAGGGTGGTT
Pparg	Forward	ATGGGTGAAACTCTGGGAGA
	Reverse	CTTGTGAAGTGCTCAGC
Scd1	Forward	CGTCTGGAGGAACATCATTCT
	Reverse	CAGAGCGCTGGTCATGTAGT

Srebp1c	Forward	GTACCTGCGGGACAGCTTAG
	Reverse	TCAGGTCATGTTGGAAACCA
Ctgf	Forward	CGACTGGAAGACACATTTGG
	Reverse	CAGGTCTTAGAACAGGCG
Cyr61	Forward	GAGTGGGTTTGTGATGAAGAC
	Reverse	CTTCAGTGAGCTGCCTTTTCC
Gapdh	Forward	GCTTGTCATCAACGGGAAG
	Reverse	GATGTTAGTGGGGTCTCG
Primers used for chromatin immunoprecipitation		
Target	Direction	Sequence (5' → 3')
Nos3 promoter	Forward	GGTCAGCGGGCATGAAG
	Reverse	AGCAGAGTCCTGGCCTT
Primers used for luciferase reporter construction		
Target	Direction	Sequence (5' → 3')
Nos3 promoter	Forward	AAAAAAACTCGAGGTGGGTTCAGGAAATTGAGATGA
	Reverse	AAAAAAAAAGCTTAGCAGAGTCCTGGCCTT
